# Next generation sequencing guided treatment of *ALK* tyrosine kinase inhibitor induced long survival in lung squamous cell carcinoma harboring *ROS1* gene fusions: a case report and literature review

**DOI:** 10.3389/fmed.2026.1771353

**Published:** 2026-03-06

**Authors:** Mei Liu, Fenge Li, Ning Mu, Ning Kang, Shengnan Wu, Yue Xu, Xinyi Wang, Huan Lü, Chunhua Ma

**Affiliations:** Department of Oncology, Tianjin Union Medical Center, The First Affiliated Hospital of Nankai University, Nankai University, Tianjin, China

**Keywords:** drug resistance, long survival, lung squamous cell carcinoma, molecular-targeted therapy, next-generation sequencing - NGS, *ROS1* gene fusion

## Abstract

Approximately 1–2% of non-small cell lung cancer (NSCLC) cases harbor *ROS1* gene fusions. However, lung squamous cell carcinoma (LUSC) patients with *ROS1* rearrangements remain exceptionally rare. Current targeted therapies for LUSC harboring *ALK*, *ROS1*, or *EGFR* mutations are typically guided by protocols established for lung adenocarcinoma. Here, we present a case of advanced LUSC with *ROS1* fusion who achieved prolonged survival (42 months) through sequential treatment with *ALK* tyrosine kinase inhibitor (*ALK*-TKI) and *ROS1*-TKI. Notably, this patient’s therapeutic management was critically informed by serial next-generation sequencing (NGS), demonstrating the value of precision medicine. Genomewide copy number profiles for the three clinical specimens demonstrate distinct genomic alteration patterns implying tumor genome signature changes over treatment and disease development. Laboratory immunofluorescence analysis of tumor biopsies further revealed treatment-induced modulation of tumor immune microenvironment (TIME), characterized by increased CD8 + T-cell infiltration and increased PD-L1 expression on tumor cells over treatment. Peripheral monocyte profiling of the patient post-Repotrectinib and localized radiotherapy showed 75% CD8+/CD3 + T cells, 14.2% CD4+/CD3 + T cells, 3.95% regulatory T cells (Tregs), and 38% PD-1 + CD3 + T cells. These systemic T cell dynamics mirror the immunophenotype observed in the tumor microenvironment. Furthermore, we also provide a comprehensive review of recent clinical advancements in *ALK/ROS1*-TKI for NSCLC, including mechanistic insights into TKI resistance development. This case underscores the therapeutic potential of molecular-targeted agents in LUSC and highlights the essential role of NGS-guided precision oncology.

## Introduction

Non-small cell lung cancer (NSCLC) comprises three primary histological subtypes: squamous cell carcinoma (accounting for approximately 30% of newly diagnosed cases) (1), adenocarcinoma, and large cell lung cancer. *ROS1* gene fusions predominantly occur in young, never-smoking female patients diagnosed with advanced NSCLC, with an incidence rate of 1–2% (2–4). Crizotinib is currently recommended as the first-line therapeutic option for *ROS1*-positive NSCLC (5–7). Research indicates that secondary *ROS1* G2032R point mutations represent the primary mechanism of Crizotinib resistance (8). Notably, the third-generation *ROS1* inhibitor Lorlatinib has demonstrated potent antitumor activity against the G2032R mutation in preclinical models (9, 10). Clinical evidence from multiple studies supports its efficacy in treating *ROS1* G2032R-mutated tumors, with favorable objective response rates reported (11, 12). However, the emergence of acquired resistance to Lorlatinib poses a significant clinical challenge in subsequent treatment strategies. Current understanding of resistance mechanisms to third-generation *ROS1* inhibitors remains constrained to preclinical experiments, individual case reports, and limited-scale clinical investigations, which collectively provide insufficient guidance for optimizing clinical management.

Emerging evidence indicates that *ROS1* gene fusions in patients with squamous cell carcinoma of the lung represent an exceedingly rare clinical entity, with only limited case reports documented in current literature (13–15). Next-generation sequencing (NGS) technology has revolutionized lung cancer management through its multifaceted applications in precision diagnostics, selection of targeted therapies, and therapeutic response monitoring. Notably, NGS enables comprehensive molecular profiling for resistance mechanisms, allowing detection of clinically relevant alterations such as *EGFR T790M* mutations and *MET* amplifications, which directly inform treatment modifications (16, 17). Despite these advantages, the clinical utility of NGS in lung squamous cell carcinoma remains contentious, primarily due to cost-effectiveness considerations and the historically low prevalence of targetable driver mutations in genes including *EGFR*, *ALK*, *ROS1*, and *RET*. Failure to perform NGS may consequently deprive patients of potentially life-extending tyrosine kinase inhibitor (TKI) therapies. Herein, we present a compelling case of an advanced lung squamous cell carcinoma patient harboring a *ROS1* fusion who achieved remarkable long-term survival (42 months) through sequential *ALK/ROS1*-TKI treatment guided by serial NGS monitoring. This case underscores the critical importance of implementing NGS-based molecular profiling in lung squamous cell carcinoma management. Furthermore, we synthesize recent clinical advancements in *ALK/ROS1*-TKI therapies for non-small cell lung cancer (NSCLC) and elucidate the molecular mechanisms underlying TKI resistance development.

## Case presentation

**Figure 1:**
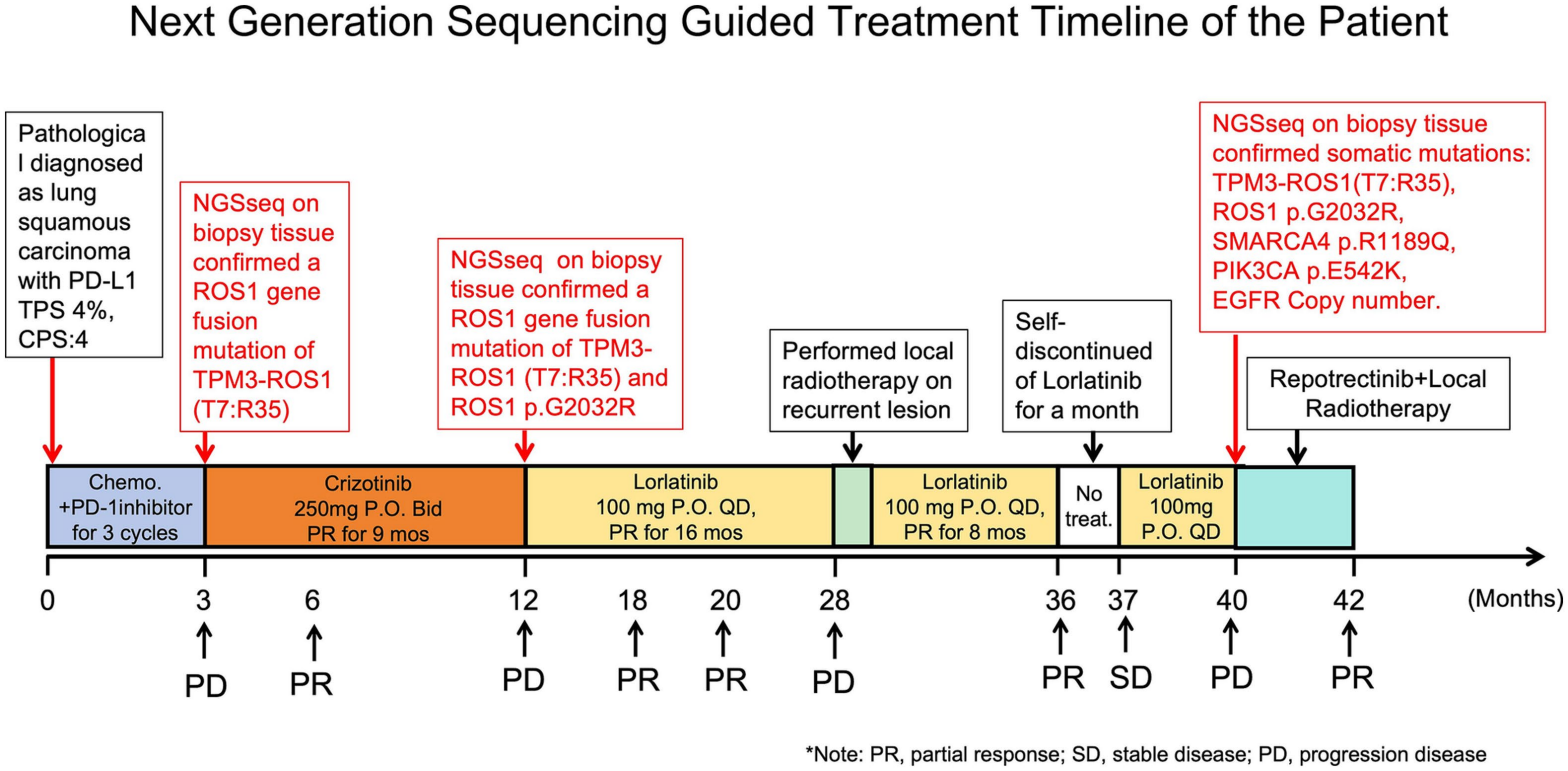
Next-generation sequencing (NGS)-guided treatment timeline in a patient with prolonged survival. The patient underwent three sequential NGS analyses, which directed personalized ALK/ROS1 tyrosine kinase inhibitor (TKI) therapies. This precision medicine approach enabled the patient to achieve prolonged progression-free survival.

**Figure 2:**
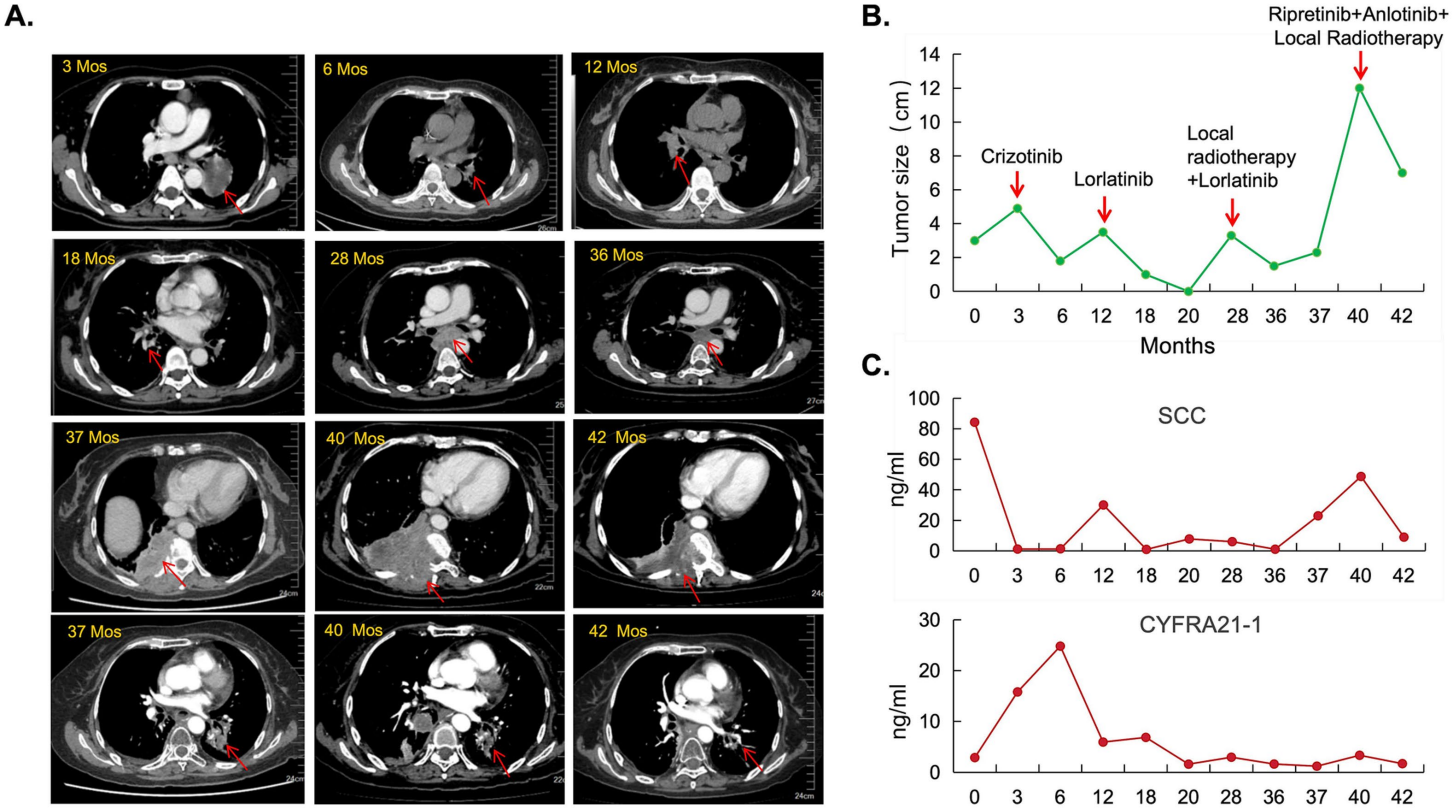
Longitudinal CT imaging and tumor dynamics during multiline therapy. **(A)** Chronological CT assessments revealed distinct tumor responses and resistance patterns across treatment phases. Initially, after 9 months of crizotinib therapy, mediastinal and right hilar lymphadenopathy (3.0 cm) emerged (at months 12). Six months post-lorlatinib initiation, the initial chest CT re-evaluation demonstrated a 46.7% tumor burden reduction. Following disease progression at 16 months post lorlatinib therapy, imaging showed progressive mediastinal lymphadenopathy with esophageal compression, left lower lobe pulmonary hilum mass enlargement, right pleural metastases with chest wall/rib/vertebral invasion, and upstream esophageal dilation. A novel combination regimen comprising repotrectinib and local radiotherapy on right chest wall induced partial response with measurable tumor regression. The patient survived for 42 months at the time of follow-up. **(B)** Quantitative tumor size kinetics across treatment phases demonstrating marked tumor shrinkage during ALK/ROS1 tyrosine kinase inhibitor (TKI) therapy. **(C)** Biomarker profiles of squamous cell carcinoma antigen (SCC0) and cytokeratin 19 fragment (CYFRA21-1) correlated with radiographic tumor control during TKI treatment phases.

A 52-year-old female patient was pathologically diagnosed with lung squamous cell carcinoma (LUSC) via CT-guided needle biopsy. She initially underwent standard first-line therapy with of chemotherapy (Paclitaxel, 175 mg/m^2^ + Cisplatin, 75 mg/m^2^) and PD-1 inhibitor (Tislelizumab, 200 mg/cycle) for 2 cycles, followed by second-line treatment with Gemcitabine (1,000 mg/m^2^) combined with cisplatin (75 mg/m^2^) and PD-1 inhibitor (Tislelizumab, 200 mg/cycle) for 1 cycle at a local hospital. Despite these interventions, the patient demonstrated radiographic progression with progressive disease confirmed by RECIST 1.1 criteria within 3 months of initiating first-line therapy, and continued tumor growth was observed during second-line treatment (Figures 1, 2A,B).

We conducted initial next-generation sequencing (NGS) analysis on her tumor tissue, identifying a *ROS1* gene fusion (*TPM3* exon 7 fused to *ROS1* exon 35; T7:R35) with a mutation rate of 26.26% and low programmed death-ligand 1 (PD-L1) expression (tumor proportion score [TPS] 4%). The patient commenced first-generation *ALK* tyrosine kinase inhibitor (TKI) Crizotinib therapy at 250 mg orally twice daily, achieving a significant objective clinical response lasting 9 months (Figures 1, 2A,B).

At 9 months post-treatment, routine chest computed tomography (CT) demonstrated disease progression characterized by multiple mediastinal and right apical lymph node metastases with interval enlargement, as well as a new obstructive lesion in the right middle lobe (Figures 2A,B). Bronchoscopic biopsy of the right main bronchus opening revealed moderately differentiated squamous cell carcinoma (p40(+), p63(+), Ki-67 40%, p53 scattered positive, CK5/6(+)). Repeat NGS confirmed persistence of the *TPM3-ROS1* fusion (mutation rate 26.26%) and acquired resistance mutation p.G2032R (13.70% mutation rate), with further reduction in PD-L1 expression (TPS < 1%). Based on these findings and the clinical progression criteria (RECIST 1.1), Crizotinib was discontinued.

The patient was subsequently initiated on third-generation *ALK*-TKI Lorlatinib at 100 mg orally once daily. Early tumor response assessment at 3 weeks showed remarkable tumor burden reduction (−46.7%) with sustained disease control for 16 months. At 16 months, chest CT revealed progressive mediastinal lymphadenopathy causing extrinsic esophageal compression and proximal esophageal dilation (Figure 2A), meeting RECIST 1.1 criteria for progressive disease (PD). Systemic staging confirmed no distant metastases beyond the thoracic cavity.

Following mediastinal lymph node-targeted radiotherapy (details omitted for brevity), the patient achieved secondary partial response (PR) with additional 8-month disease stabilization. However, temporary discontinuation of Lorlatinib occurred due to financial constraints. Subsequent CT scans demonstrated interval disease progression with left lower lobe lesion enlargement and right pleural thickening prompting re-initiation of Lorlatinib therapy. Despite retreatment, the patient developed right thoracic pain and CT follow-up revealed progressive left lower lobe tumor growth, right pleural metastases with chest wall/rib/vertebral invasion, and interval regression of mediastinal lymphadenopathy (Figure 2A). Histopathological confirmation of right chest wall metastatic lesion confirmed squamous cell carcinoma consistent with lung origin.

The third concurrent comprehensive genomic profiling using next-generation sequencing (NGS) revealed multiple somatic alterations, including a *ROS1* gene fusion (*TPM3-ROS1*, T7:R35) with a variant allele frequency (VAF) of 28.04%, a *ROS1* p.G2032R solvent front mutation (VAF 11.78%), a *SMARCA4* p.R1189Q mutation (VAF 13.57%), a *PIK3CA* p.E542K hotspot mutation (VAF 5.15%), and *EGFR* gene copy number gain (CN: 2.7). Following these findings, the patient received Repotrectinib (dosing: 160 mg orally once daily) combined with palliative radiotherapy to right chest wall metastases for symptom control. Subsequent chest CT imaging demonstrated an on-target therapeutic response with significant tumor shrinkage at 2 months (RECIST 1.1 partial response; Figures 2A,B).

**Figure 3:**
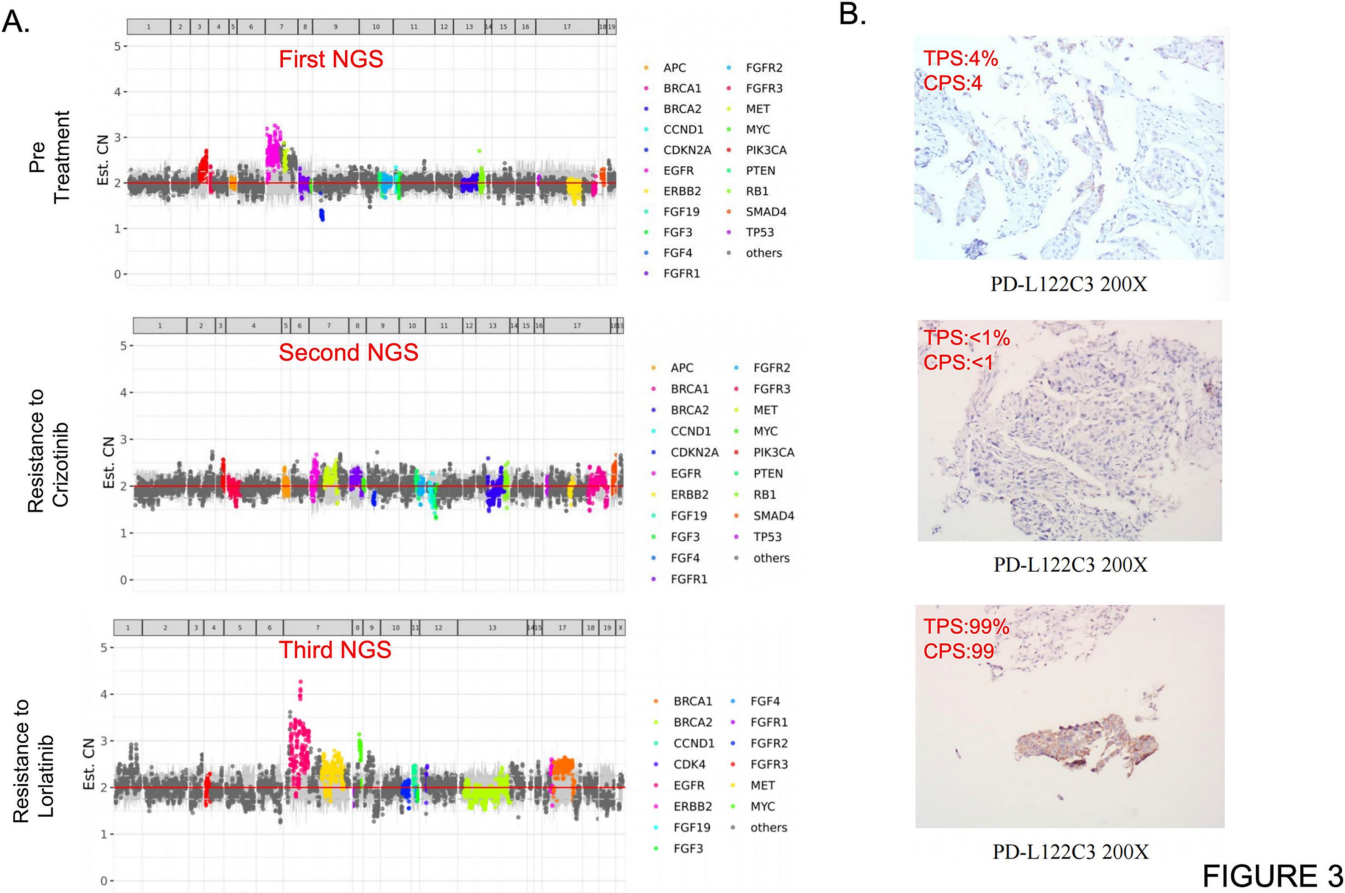
Next-generation sequencing (NGS) test results and PD-L1 expression profiles of the three clinical samples. **(A)** Genome wide copy number profiles for the three clinical specimens demonstrate distinct genomic alteration patterns. Each data point represents a genomic interval, with color-highlighted points indicating regions exhibiting clinically significant copy number variations (CNVs). The horizontal axis displays chromosomal coordinates (chromosome number and base pair position), while the vertical axis shows normalized copy number values derived from NGS analysis (red horizontal line indicates diploid copy number baseline). Notably, the observed copy number alterations may be attenuated by the presence of normal cell admixture in clinical specimens, thereby potentially underestimating the true clonal copy number variations in tumor cells. **(B)** Programmed death-ligand 1 (PD-L1) expression was significantly upregulated in three distinct tumor tissue specimens following ALK/ROS1 tyrosine kinase inhibitor (TKI) treatments.

**Figure 4:**
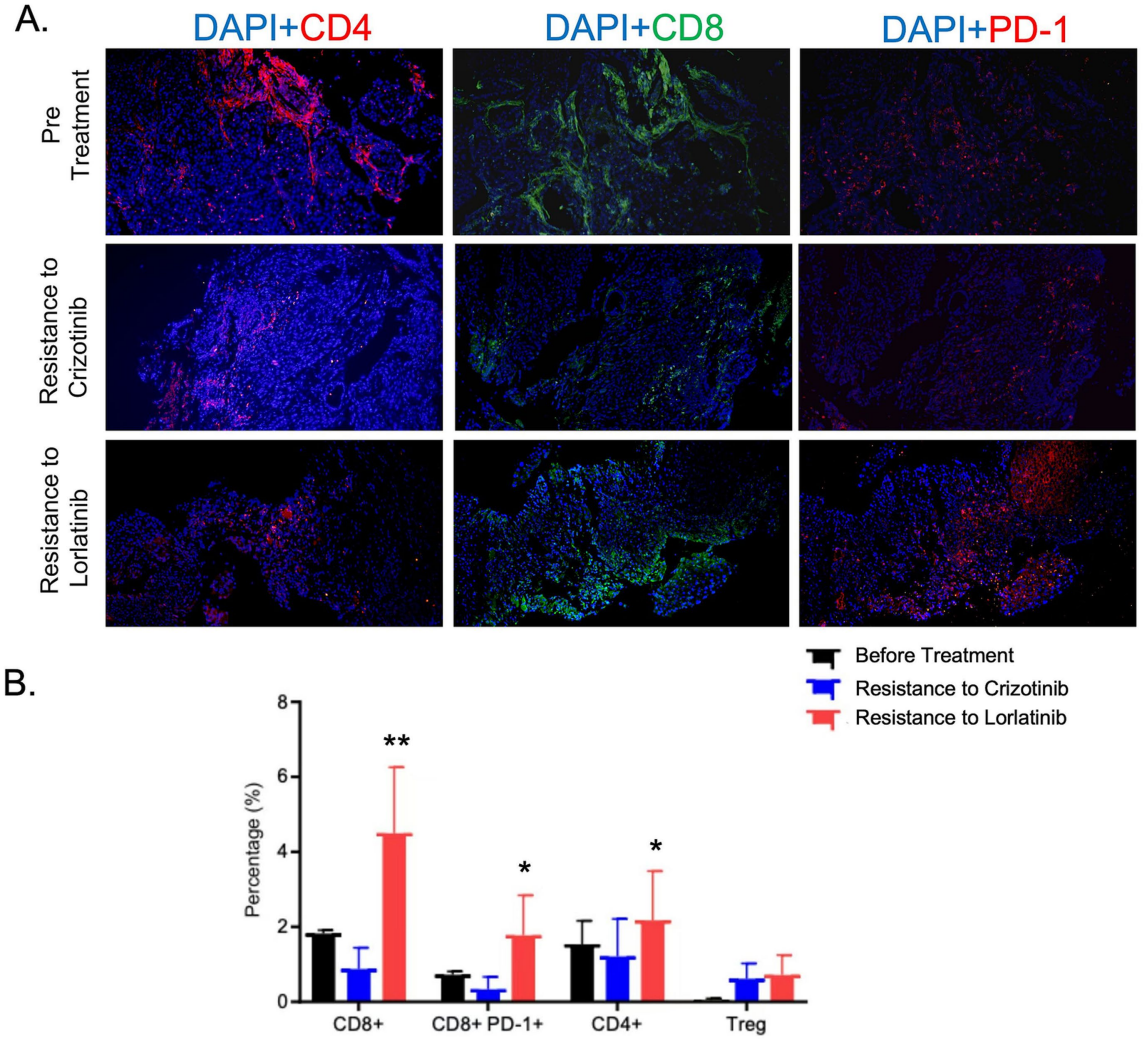
Immunofluorescence characterization of tumor-infiltrating lymphocytes in three biopsy specimens. **(A)** Representative immunofluorescence micrographs demonstrating specific staining patterns for individual lymphocyte markers. **(B)** Quantitative flow cytometry analysis revealed statistically significant increases in both CD8 + T cells and CD8 + PD-1 + T cell subsets following targeted therapy (* *p* < 0.05, ** *p* < 0.01).

Concurrently, serum tumor biomarkers exhibited dynamic changes synchronous with tumor burden. The level of squamous cell carcinoma antigen (SCC) decreased, consistent with the changes in tumor burden. However, the fluctuations in cytokeratin 19 fragment (CYFRA21-1) did not appear to significantly correlate with the clinical course, possibly due to its expression being dependent on the squamous epithelial differentiation characteristics of the tumor and its relatively lower sensitivity in *ROS1*-driven cancers (Figure 2C). Apart from the currently monitored SCC and CYFRA21-1, circulating tumor DNA (ctDNA), serum carcinoembryonic antigen (CEA) and soluble programmed death-ligand 1 (sPD-L1) can also be utilized for disease monitoring in future studies of LUSC patients. Genomewide copy number profiles for the three clinical specimens demonstrate distinct genomic alteration patterns implying tumor genome signature changes over treatment and disease development (Figure 3A). Programmed death-ligand 1 (PD-L1) expression was significantly upregulated in three distinct tumor tissue specimens following *ALK/ROS1* tyrosine kinase inhibitor (TKI) treatments (Figure 3B). Longitudinal immunofluorescence analysis of tumor biopsies further revealed treatment-induced modulation of tumor immune microenvironment (TIME), characterized by increased CD8 + T-cell infiltration and increased PD-L1 expression on tumor cells over treatment (Figures 4A,B). Peripheral monocyte profiling of the patient post-Repotrectinib and localized radiotherapy showed 75% CD8+/CD3 + T cells, 3.95% regulatory T cells (Tregs), and 38% PD-1 + CD3 + T cells. These systemic T cell dynamics mirror the immunophenotype observed in the tumor microenvironment (Supplementary Figures 1A,B).

## Discussion and literature review

In this case report, we present a 52-year-old female patient diagnosed with advanced lung squamous cell carcinoma (LUSC) harboring a *TPM3-ROS1* gene fusion—a remarkably low-prevalence molecular alteration in LUSC. The administration of Crizotinib (a first-generation *ROS1* tyrosine kinase inhibitor) achieved marked tumor regression within 3 months, thereby establishing *ROS1* fusions as actionable therapeutic targets in metastatic LUSC. This clinical response corroborates the current international guidelines recommending Crizotinib as first-line therapy for advanced non-small cell lung cancer (NSCLC) with *ROS1* gene rearrangements (18–20).

Notably, lung squamous cell carcinoma (LUSC) exhibits distinct biological behaviors and tumor microenvironment characteristics compared to lung adenocarcinoma (LUAD), with traditionally lower responsiveness to targeted therapies (21). However, the durable clinical benefit observed in this LUSC patient following crizotinib treatment aligns with the median progression-free survival (mPFS: 6–19 months) reported in *ROS1*-rearranged LUAD cohorts treated with crizotinib (22). This clinical observation suggests that the therapeutic significance of *ROS1* fusion status may extend beyond conventional histopathological classifications, offering pivotal implications for molecular diagnostic protocols in clinical oncology. Given the potential for actionable genomic alterations in LUSC—a subtype historically characterized by limited druggable targets—routine comprehensive genomic profiling using next-generation sequencing (NGS) should be implemented to identify targetable driver mutations such as *ROS1* fusions.

The patient developed progressive mediastinal and right hilar lymphadenopathy with metastatic features, indicating disease progression (PD) and acquired resistance to Crizotinib after 9 months of treatment. A second next-generation sequencing (NGS) analysis identified a novel *ROS1* G2032R mutation. Notably, secondary *ROS1* G2032R mutations have been established as the predominant molecular mechanism underlying Crizotinib resistance (23, 24). This glycine-to-arginine substitution at residue 2032 within the *ROS1* kinase domain induces steric hindrance that obstructs drug binding, thereby conferring resistance to *ROS1* kinase inhibition. Importantly, the G2032R mutation has also been shown to upregulate *TWIST1* expression and promote epithelial-mesenchymal transition (EMT), further contributing to Crizotinib resistance (25). Upon switching to Lorlatinib—a third-generation *ROS1* inhibitor the patient achieved a rapid 46.7% reduction in tumor burden, resulting in sustained disease control for 16 months. These clinical findings corroborate preclinical evidence demonstrating the superior inhibitory activity of Lorlatinib against the *ROS1* G2032R mutation. Comparative studies have revealed that Lorlatinib exhibits significantly enhanced cytotoxicity against G2032R mutant cell lines *in vitro* and superior antitumor efficacy *in vivo* compared to Crizotinib (9, 10). Clinical trials further confirm improved disease response rates in patients with G2032R mutations treated with Lorlatinib versus Crizotinib (11, 12). Collectively, these data underscore the pivotal role of Lorlatinib in overcoming Crizotinib resistance.

This patient achieved an exceptional survival benefit of 25 months with *ALK/ROS1* TKI therapies, surpassing the median progression-free survival (mPFS) of 17.9 months reported in prior clinical studies (26). Several factors potentially contributed to this prolonged response in advanced lung squamous cell carcinoma: First, serial NGS provided critical molecular evidence enabling precision targeting with Lorlatinib. Second, the absence of additional resistance mutations during early Lorlatinib treatment suggests limited clonal evolution of resistant tumor cells. Third, localized radiotherapy effectively controlled regional disease progression, thereby prolonging the efficacy of systemic therapy. Notably, a brief one-month treatment interruption followed by Lorlatinib rechallenge resulted in rapid chest wall, rib, and thoracic vertebral metastases, indicating dynamic genomic evolution. Subsequent NGS analysis of progressive disease biopsy specimens revealed multiple somatic alterations: the original *TPM3-ROS1* fusion allele frequency increased to 28.04%, *ROS1* G2032R mutation frequency reached 11.78%, and novel alterations emerged including *SMARCA4 p.R1189Q, PIK3CA p.E542K, EGFR* copy number amplification, and marked PD-L1 overexpression (99% tumor proportion score [TPS]). The underlying mechanism may be explained by three interrelated factors: First, during lorlatinib treatment, sensitive tumor clones are suppressed, while resistant subclones remain dormant due to competitive fitness disadvantages. Upon treatment discontinuation, the selective pressure is lifted, allowing resistant subclones to rapidly expand. Concurrently, the rate of random somatic mutations increases, accelerating mutational accumulation. Second, lung squamous cell carcinoma (LUSC) is characterized by inherent genomic instability, with higher levels of chromosomal fragmentation, copy number variations, and gene mutation rates compared to lung adenocarcinoma, along with significantly impaired DNA repair mechanisms (27). Combined with the high Ki-67 index (40%) observed in this case, these features provide a permissive genetic and proliferative environment for the rapid emergence of multiple mutations within a short timeframe. Third, cumulative genomic damage from prior therapies further exacerbates genomic instability. Previous lines of chemotherapy and radiotherapy induce DNA damage in tumor cells and impair DNA repair capacity, thereby amplifying genomic instability and facilitating the swift development of resistance-associated mutations following treatment cessation. These findings suggest Lorlatinib resistance involves multifaceted mechanisms including novel driver mutations, bypass pathway activation, tumor immune microenvironment remodeling, and systemic immune transformation (Figures 4A,B).

The *SMARCA4* gene encodes a core subunit of the SWI/SNF chromatin remodeling complex. Loss-of-function *SMARCA4* mutations induce chromatin structural abnormalities that modulate tumor cell sensitivity to targeted therapies. Emerging evidence indicates that *SMARCA4* inactivation can restore sensitivity to tyrosine kinase inhibitors (TKIs) in non-small cell lung cancer (NSCLC), including *ALK*-TKI and *ROS1*-TKI (28, 29). The *PIK3CA E542K* mutation activates the *PI3K-AKT–mTOR* pathway independently of *ROS1* signaling, thereby facilitating tumor cell proliferation through bypass activation—a well characterized resistance mechanism in NSCLC previously observed in *EGFR*-TKI and *ALK*-TKI resistance (30–32). *EGFR* copy number amplification may attenuate Lorlatinib’s inhibitory effects through enhanced *EGFR* signaling and cross-compensatory interactions with the *ROS1* pathway (33). The remarkable PD-L1 over expression (99% TPS) observed during progression suggests tumor immune escape via adaptive immune evasion mechanisms, consistent with previous reports of PD-L1 up regulation following *ALK/ROS1*-TKI treatment (34). The molecular mechanisms by which *ALK/ROS1*-TKI regulate tumor and systematic PD-L1 and PD-1 expressions are needed to be further explored.

It is noteworthy that the patient demonstrated another rapid tumor shrinkage when administered the combination of *ROS1* tyrosine kinase inhibitor (TKI) Repotrectinib and localized radiotherapy. As a novel, broad-spectrum *ROS1* inhibitor—Repotrectinib, with its compact, macrocyclic structure, can effectively overcome steric hindrance caused by resistance mutations and demonstrates potent inhibitory activity against *ROS1* solvent-front mutations, including G2032R (35, 36). This combinatorial approach appears to demonstrate synergistic activity in controlling tumor progression. However, the precise contribution of Repotrectinib to disease control remains to be determined, necessitating prospective follow-up studies on Repotrectinib monotherapy over the coming months. Our findings underscore the critical therapeutic value of next-generation sequencing (NGS) guidance, particularly in patients with lung squamous cell carcinoma who have developed resistance to multiple lines of treatment. Furthermore, the immunomodulatory effects of *ALK*-TKI and *ROS1*-TKI treatments on the tumor microenvironment warrant comprehensive investigation. Such research could establish the mechanistic basis for subsequent immunotherapy strategies in patients who have developed resistance to these targeted therapies.

To elucidate the marked increase in intra-tumoral PD-L1 expression observed in the patient upon developing resistance to Lorlatinib, we conducted a comprehensive analysis of dynamic T-cell subset alterations across distinct tumor progression stages (pre-treatment, Lorlatinib-resistant, and Crizotinib-resistant phases) using multiplex immunofluorescence staining (Figures 4A,B). Our findings demonstrated that the proportions of both CD8 + T cells and CD4 + T cells were significantly elevated in Lorlatinib-resistant tumor samples compared to pre-treatment and Crizotinib-resistant specimens, accompanied by a notable enrichment of CD8 + PD-1 + exhausted T cells. These data suggest that the development of Lorlatinib resistance correlates with enhanced infiltration of effector T cells and a pronounced immunosuppressive tumor microenvironment. Notably, this phenotypic shift may be attributed to localized radiotherapy, which induces rapid tumor cell death and releases abundant tumor-associated antigens, thereby promoting T-cell recruitment to the tumor site.

Importantly, the potential role of Lorlatinib in enhancing tumor lymphocyte infiltration remains to be validated through rigorous *in vivo* and *in vitro* investigations. This necessity arises due to the scarcity of existing reports regarding tumor PD-L1 expression dynamics following the development of *ALK*-TKI or *ROS1*-TKI resistance in lung cancer patients. Furthermore, the clinical benefit of anti-PD-L1 therapy for patients exhibiting *ROS1*-TKI resistance requires systematic evaluation through robust clinical evidence. As targeted therapy continues to advance, particularly in the context of Lorlatinib resistance, emerging evidence suggests that multi-pathway mutations may significantly modulate the tumor microenvironment through alternative mechanisms such as *PI3K-AKT* pathway activation. This phenomenon could potentially counteract the therapeutic advantages of immunotherapy, despite the observation that regulatory T cell (Treg) populations do not demonstrate significant expansion in these scenarios.

## Clinical implications and study limitations

For patients with advanced lung squamous cell carcinoma (LUSC), routine molecular profiling should encompass driver gene testing (including *ROS1* fusion detection) and PD-L1 expression assessment even in the absence of traditional favorable factors for targeted therapy such as never-smoking history. Particularly following acquired resistance to sequential treatment lines, integrated analysis combining comprehensive genomic profiling (utilizing tissue-based next-generation sequencing) and immune microenvironment characterization is warranted to capture dynamic alterations in tumor molecular landscapes and immune status, thereby preventing missed opportunities for precision therapies. As tissue-based assays are frequently constrained by limited sample availability during treatment, the supplementary role of liquid biopsy (ctDNA-NGS) may be particularly valuable for patients in whom tissue biopsies are difficult to obtain. Non-invasive, dynamic monitoring of driver gene alterations, the emergence of resistance mutations, and novel variants can be achieved through ctDNA-NGS, providing timely evidence for therapeutic adjustments. Furthermore, we conducted next-generation sequencing using Illumina platforms, which employ short-read sequencing technology and necessitate fragment assembly. A more accurate third-generation sequencing technology, which utilizes platforms such as Oxford Nanopore and PacBio, can perform single molecule long-read sequencing, allows real-time single-molecule sequencing, shows superior performance in detecting structural variations and assembling complex genomes, and is recommended for future clinical applications.

However, this study has several inherent limitations as a single-case report. First, the generalizability of our findings requires further validation through multi-center trials with larger patient cohorts. Second, the current follow-up duration of Repotrectinib combined with radiotherapy remains insufficient to comprehensively evaluate long-term efficacy endpoints, safety profiles, and longitudinal impacts on tumor immune microenvironment dynamics.

## Conclusion

This case report provides critical insights into the clinical management of lung squamous cell carcinoma with *ROS1* gene fusions. Our findings underscore the necessity for routine molecular testing in advanced LUSC patients particularly emphasizing *ROS1* fusion detection to identify actionable therapeutic targets beyond traditional clinic opathological predictors. We demonstrate that dynamic monitoring of tumor evolution through sequential genomic and immunological assessments during targeted therapy is essential for timely treatment adaptation and resistance mitigation. Future research should prioritize mechanistic studies on resistance development in this rare molecular subtype, while developing rational combination strategies that integrate targeted therapies with immune modulation to optimize clinical outcomes.

## Data Availability

The raw data supporting the conclusions of this article will be made available by the authors, without undue reservation.

## Supplementary material

SUPPLEMENTARY FIGURE 1 Flow cytometry analysis on peripheral monocyte in the patient post-repotrectinib and localized radiotherapy regimen showed 75% of CD8+/CD3+ T cells, 3.95% of regulatory T cells (Tregs), and 38% of PD-1+CD3+ T cells **(A,B)**. The compositions in T cells throughout the system are consistent with what we observed in the tumor micro-environment.
