# Supplementary figures and images for: Next generation sequencing guided treatment of ALK tyrosine kinase inhibitor induced long survival in lung squamous cell carcinoma harboring ROS1 gene fusions: a case report and literature review
Source: Front Med (Lausanne). 2026 Mar 6;13:1771353. doi: 10.3389/fmed.2026.1771353 (PMC13002401; doi:10.3389/fmed.2026.1771353)

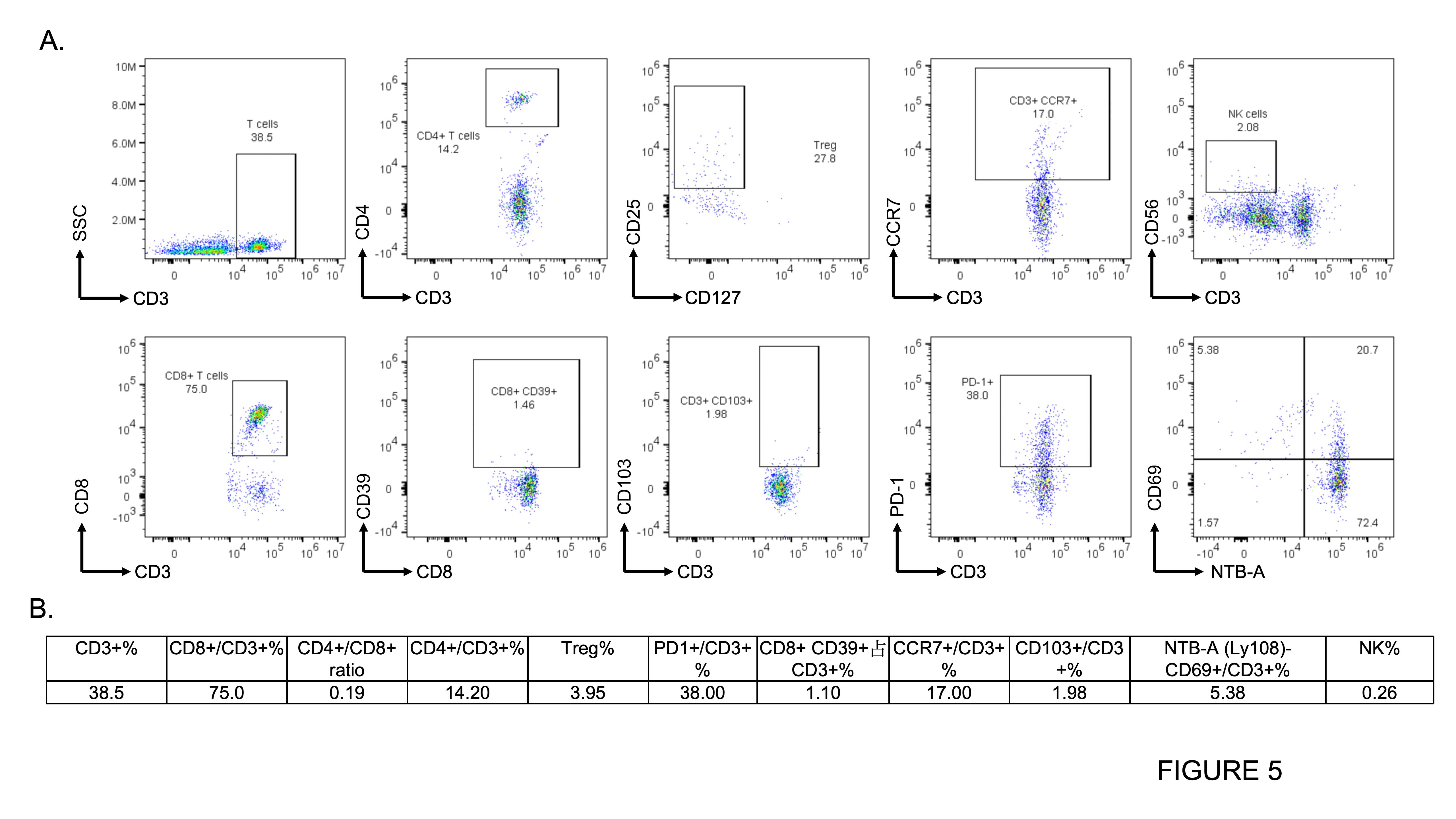

Supplement: Supplementary file 1 [file Image_1.jpeg]
